# Modeling of land suitability for surface irrigation using analytical hierarchy process method in Belessa Districts, northwestern Ethiopia

**DOI:** 10.1016/j.heliyon.2023.e13937

**Published:** 2023-02-28

**Authors:** Azemeraw Wubalem

**Affiliations:** University of Gondar College of Natural and Computational Sciences, Department of Geology, Gondar, Amhara, Ethiopia

**Keywords:** Land suitability, Analytical hierarchy process, Irrigation, GIS

## Abstract

The objective of this paper was to determine whether the land in Belessa is suitable for surface irrigation. For this, a GIS-based Analytical hierarchy process method was applied. Eight factors/parameters such as soil type, soil depth, soil texture, soil drainage, slope, distance from a water supply, and land cover were used to evaluate the suitability of the area for surface irrigation. The weight of each parameter was calculated using an 8 × 8 pairwise comparison matrix. Then, the land suitability map for surface irrigation was produced by adding weighted parameters using the weighted overlay method under ArcGIS 10.3 software. The land suitability map was divided into four categories: highly suitable (S_1_), moderately suitable (S_2_), marginally suitable (S_3_), and unsuitable (S_4_ or N_1_). The results show that 12.2% of the study area is not suitable (S4 or N1) for surface irrigation, whereas 13.9% of the study area is highly suitable (S1) for surface irrigation. Marginally (S_3_) and moderately suitable (S_2_) classes covered 26.9%, and 46.9% of the study area, respectively. The accuracy of the model was evaluated by overlaying preexisted irrigation scheme in the study area, which falls under highly and moderately suitable area, confirms that the model is accurate. Therefore, this result will be important to increase crop production in the study area, by implement surface irrigation in highly and moderately suitable lands.

## 1 Introduction

Agriculture is well documented in Ethiopia as the primary driver of the country's economic growth and long-term food security [[1], [2], [3], [4], [5]]; Labiso & Yagaso 2021). It directly supports about 85% of the population's livelihoods and 43% of the country's gross domestic product (GDP) [6]. However, it is traditionally rain-fed and subsistence-based, and it is affected by the spatial and temporal unpredictability of rainfall in various parts of the country [2,3,7,8]; Hagos et al., 2022). This resulted in recurrent crop failures and droughts, which have had a great impact on agricultural output and food availability for the country's rapidly rising population. Only 190 000 ha (5.3%) of Ethiopia's 3.7 million hectares of irrigable land have been irrigated [[2], [3], [4],[5], [9]]; Labiso & Yagaso 2021). Because the irrigation systems are still in their infancy and have limited development to help the country's agriculture sector thrive [1,[3], [5], [10]]; Labiso & Yagaso 2021).

The study area (Belessa) is one of Ethiopia's Districts in which rain-fed agriculture is the only viable option for food crop production. Food insecurity is common in Belessa because of the temporal and spatial variability of rainfall. In order to quadruple crop production during the dry season and transform low-productivity rain-fed agriculture into an effective and efficient irrigation support agriculture system, it is crucial to use surface irrigation from continually flowing river water [1,[4], [10], [11]]; Labiso & Yagaso 2021). Although the study area is affected by drought due to unpredictability rainfall variability, no study has been done yet in this area regarding land suitability assessments for surface irrigation. Therefore, a land suitability study for surface irrigation in this area is critical to support rain-fed agriculture through irrigation, which will aid in poverty alleviation. It can make a significant contribution to some of the most difficult sustainable development goals (SDGs), such as SDG 1 for ending poverty, SDG 2 for reducing hunger, achieving food security, improving nutrition, and promoting sustainable agriculture, and SDG 3 for promoting healthy lives and well-being (Ahmad et al., 2020; Ahmad et al., 2021).

Land suitability analysis can be carried out using the GIS-based analytical hierarchy process (AHP) technique, which considers a set of criteria or characteristics, such as physical properties of soils, topographic condition, proximity to a water source, and land use/land cover [[12], [13], [14], [15]]; Kasaye et al., 2019). As stated by Ref. [16]; Multi-Criteria Land Suitability Analysis (MCLSA) method, is used to make optimal decisions to select suitable land for surface irrigation. It can work with numerical algorithms that determine solution suitability based on input criteria and weights, as well as some mathematical or logical means of deciding trade-offs when conflicts exist. The weights for each factor/parameter datasets will be calculated using pairwise comparison method, and the results can be overlaid using GIS-based multi-criteria analysis techniques [17].

This study's main goal is to assess and identify the suitability of the study area's (Belessa Districts) land for surface irrigation using the GIS-based Analytical Hierarchy Process (AHP) method. The result will be used as guideline by irrigation and agricultural sector and land use planning officers for irrigation land development of the study area.

## 2 Study area

**Fig. 1:**
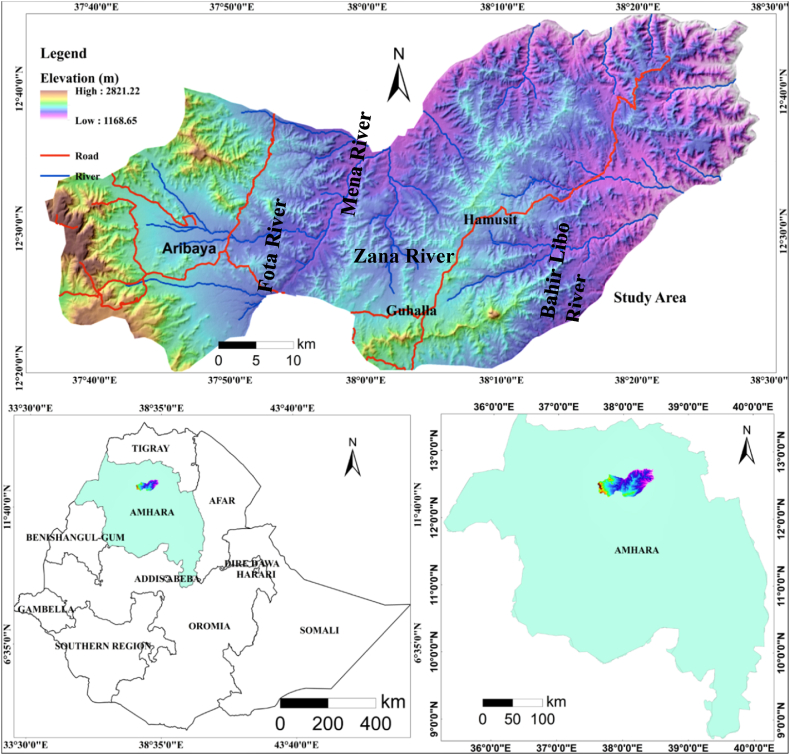
Location map of the study area.

The study area is located in Ethiopia's northwestern highlands, in the central Gondar zone of Amhara Regional State. The study area covers the Districts of East and West Belessa. They can be accessed by asphalt and gravel roads. The study area's minimum and maximum altitudes are 1,169 m in the river gorge and 2,821 m in the hills and plateau areas (Fig. 1). A study area is characterized by a lot of permanent big rivers such as Fota, Mena, Zana, and Bahir Libo Rivers. The study area is characterized by a ridge, rock, hill, plateau, deep River canyon, and delicate slope. Most of the population in the study region earns their living from agriculture.

## 3 Materials and methods

### 3.1 Data collection

Data collection and evaluation are the first important steps in land suitability mapping for surface irrigation. The soil and land use land cover data were collected from the Ethiopian Ministry of Water, Irrigation, and Energy (MoWIE), harmonized world soil database (HWSD) V: 12, and the USGS website. The SRTM DEM was downloaded from the USGS website of https://www.earthexplorer.usgs.gov (Table 1). From soil data, the physical parameters including soil depth, soil drainage, soil texture, and soil type were developed. The slope gradient and elevation are types of topographic parameters, which were extracted from the SRTM DEM (30 m resolution). The Euclidian distance was used to calculate the distance from the river. The land use and land cover map of the study area was prepared using ERDAS and GIS 10.3.

**Table 1:** Data used and sources.

Data type	Data format	Resolution	Data sources	Drive parameter map
Soil map	Vector	1:250 000	Ethiopian Ministry of Water, Irrigation, and Energy (MoWIE) and Harmonized World Soil Database (HWSD) V:12	Soil type, soil depth, soil drainage, and soil texture
DEM	Raster	30 m	USGSportal: https://www.earthexplorer.usgs.gov	Slope, elevation, and distance to a river
Land use land cover data	Raster	30 m	Ethiopian Ministry of Water, Irrigation, and Energy (MoWIE) and USGS portal: https://www.earthexplorer.usgs.gov	Land use land cover

### 3.2 Analytical hierarchy process method (AHP)

Saaty first established the AHP approach in 1978, and it is still a commonly used multicriteria decision analysis (MCDA) method [18]; 1979, [19]. The popularity of AHP comes from its ease of understanding, execution, and interpretation of outcomes by both modelers and decision-makers (Assefa et al., 2018; Woltersdorf et al., 2018). It enables researchers to depict a wide range of human decision-making logic, taking into account both qualitative and quantitative data, and combining them by dissecting difficult situations into systematic hierarchies that rank alternatives according to various criteria (Assefa et al., 2018; Woltersdorf et al., 2018). As a result, the AHP has been used to solve a wide range of water and environmental challenges including water resource allocation (Zhang et al., 2019), selection (Grum et al., 2016), feasibility evaluation (Cozzi et al., 2015), and priority and ranking (Hassani et al., 2019; Zhang et al., 2019). It's worth noting that well-founded goals are critical for defining the issue and should be incorporated in any decision-making process (Turskis et al., 2019d). As a result, one of the most important responsibilities for stakeholders using MCDA approaches is determining the importance of criterion. For this reason, the AHP approach is the most extensively utilized method (Zavadskas et al., 2016). The procedure of determining site suitability for surface irrigation involves two main steps in this study. The influential geographical decision criteria and sub-criteria were assessed in the first step. Then, utilizing the AHP technique, a GIS-MCDA model was created to solve the spatial decision-making process. Geospatial data and ArcGIS 10.3 software were used to implement the MCDA approach.

### 3.3 Defining criteria and sub-criteria

In AHP analysis, defining criteria and sub criteria is the second step following the goal of the AHP analysis. A factor is a criterion that can increases or decreases the suitability of a certain alternative for the activity being considered. The practice of evaluating and classifying specific portions of land based on their appropriateness for specific applications is known as land suitability classification [11]. This type of land suitability analysis is critical for development because it provides key information about the potential land use options. As stated by Ref. [11]; land suitability is the fitness of a given type of land for an outlined use [11,20]. developed land suitability classes of highly suitable to not suitable lands (Table 3 [11]). These classes are generally classified into suitable and not suitable (S_4_ or N). In 1976 and 1983, FAO further classified the land into three and two classes, respectively. These are highly suitable (S_1_), moderately suitable (S_2_), marginally suitable (S_3_), temporarily not suitable (S_4_/N_1_), and permanently not suitable (S_5_/N_2_). As indicated in Table 2, different researchers at different times have classified parameters to evaluate the suitability of land for surface irrigation. In this study, land suitability factors for surface irrigation were classified using Table 2, Table 3 guidelines. For the assessment of appropriate land for surface irrigation, eight primary influential criteria as determinants were selected based on the current literature, agricultural history, data availability, and expert opinions (Bagherzadeh and Gholizadeh 2016; [2,[3], [5], [21], [22], [23]]). 1) Slope; 2) land use land cover; 3) elevation; 4) soil attributes (depth, texture, type, and drainage); and 5) distance to rivers are the eight factors used in the analysis. Different sub-criteria are used to examine each of these factors individually. Sub-criteria were treated differently depending on whether they were discrete or continuous.

**Table 2:** Significance of physical factors for surface irrigation.

Parameters	Parameters classes	Degree of significance	Sources
Drainage	well moderately well imperfectly poor excessive	optimum moderate marginal not ideal for upland crops as little significance	[24] [3]
Soil depth (cm)	<10 10–50 50–100 >100	very low low marginal optimum	[25] [3]
Soil type	chromic luvisols humic nitosols eutric vertisols haplic calcisols rendzic leptosols lithic leptosols	optimum moderate marginal low very low very low	FAO and UNESCO 1988 [3]
Texture classes	sandy loam loamy sandy loam silt loam	marginal low optimum moderate	[26] [25] [3]
Euclidean distance (km)	0–5 5–10 10–20 >20	optimum moderate marginal low	[25] [3]
LULC	rangelands farmland dispersed forest settlement bush bare land	moderate optimum low not optimal marginal not optimal	[25] [3]
Slope (%)	0–2 2–5 5–8 >8	optimum moderate marginal low	[25] [26] [27] [3]

**Table 3:** Land suitability classification structures [11] and 1981).

Class	Description
S_1_ Highly Suitable	Land without significant limitations. This land is the best possible and does not reduce productivity or require increased inputs.
S_2_ Moderately Suitable	Land that is clearly suitable but has limitations that either reduce productivity or require an increase of inputs to sustain productivity compared with those needed on S_1_ land
S_3_ Marginally Suitable	Land with limitations so severe that benefits are reduced, and/or the inputs required to sustain production need to be increased so that this cost is only marginally justified.
N_1_ Currently Not Suitable	The land has limitations that may be surmountable in time, but which cannot be corrected with existing knowledge at a currently acceptable cost; the limitations are so severe as to preclude successful sustained use of the land in the given manner
N_2_ Permanently Not Suitable:	The land has limitations that appear as severe as to preclude any possibilities of successful sustained use of the land in the given manner.

#### 3.3.1 Topographic parameters (slope and elevation)

Elevation and slope maps were generated from the Shuttle Radar Topography Mission (SRTM) digital elevation model (DEM) (Moore and Hansen 2011; Gorelick 2013). The slope and elevation of the study area were divided into different suitability classes using the [28] guideline. The slope is one of the parameters in land suitability analysis for surface irrigation, which may affect land preparation, irrigation operation, moisture content, production costs, soil depth, erosion, and method of irrigation [3,26]. The slope was classified into four suitable classes 0–2%, 2–5%, 5–8%, and >8% [3,11,26]. When the slope is steep, surface irrigation operation will be hard and the soil erosion rate will be high. This results in less agricultural productivity.

#### 3.3.2 Soil parameters

As stated by Refs. [26,29]; the soil is a crucial factor to work out the suitability of land for sustainable surface irrigation and agriculture. Productivity, irrigation operations, and irrigation development can all be impacted by the character of the soil (Kasaye et al., 2019). The physical characteristics of soil are often evaluated to work out soil suitability for irrigation [26]. The suitability of soil for irrigation and agriculture are depend on soil fertility, moisture content, depth, and drainage [26]. For this study, physical soil factors (soil type, soil drainage, soil depth, and texture classes) are considered as the primary soil factors.

#### 3.3.3 Land use land cover parameters

Land use is one of the important parameters in land suitability analysis for surface irrigation. The land use map was generated from Landsat 8 satellite image. It had classified into four suitable classes of highly suitable (S_1_), suitable (S_2_), marginally suitable (S_3_), and not suitable (S_4_). Grassland is considered to be just somewhat suitable (S_2_), while cropland was rated as extremely suitable (S_1_). Woodland/Shrub/bush was classified as marginally suitable (S_3_) because it required initial investment for land preparation [3]. The forest, barren land, a water body and settlements are deemed unsuitable (S4).

#### 3.3.4 Distance to streams/rivers

Evaluation and determination of the distance to the water source are among the foremost important steps in land suitability analyses for surface irrigation. Distance from the water source (permanently flowing river) was generated using Euclidean distance in ArcGIS which was classified into <5 km (S_1_), 5–10 km (S_2_), 10–20 km (S_3_), and >20 km (S_4_).

### 3.4 Developing the AHP model

One of the semi-quantitative methods that will be used to determine land suitability for surface irrigation is named AHP. AHP may be a structured tool that helps to research difficult decisions supported by mathematics and psychology [30]; Nguyen et al., 2015; [31]. In the GIS-MCDA framework, the AHP technique [18] was implemented, in which a hierarchical mechanism was used to rank criteria and alternatives according to their value (Cozzi et al., 2015). Pairwise comparisons of the criteria are used in the AHP to assist decision-makers in determining the relative importance of the criteria. The weights and rankings of the criteria for the GIS-MCDA model were determined through this method. The following stages show how the AHP model for this investigation was created.

#### 3.4.1 Formulating hierarchy

**Fig. 2:**
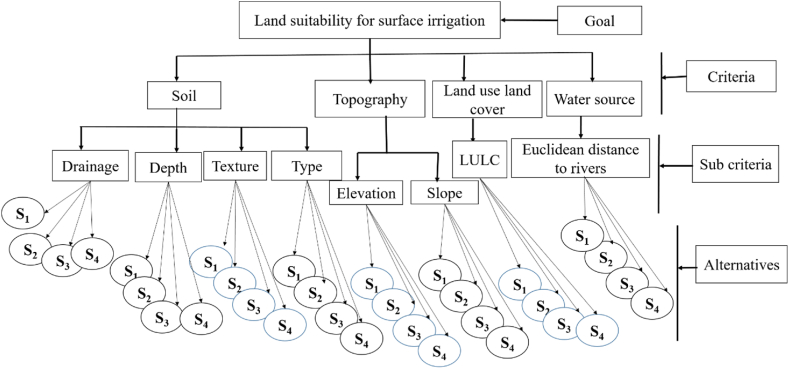
Analytical hierarchy structure.

After defining all of the chosen criteria and analyzing sub-criteria, the hierarchy structure was created. To arrange the complex multi-criteria problem on a number of levels, the hierarchy structure was articulated using the AHP method. A goal, major criteria, sub-criteria, and options are all included in the decision hierarchy. The hierarchy's first level corresponds to the problem's overall goal, the second level to the criteria, and the third level to the sub-criteria [19]. The key criteria and sub-criteria for selecting eligible areas for irrigation were outlined in the first and second stages of the hierarchy, as shown in Fig. 2, depending on their priority (explained above).

#### 3.4.2 Weighting the criteria and sub-criteria

The weights of each criterion and sub criteria are calculated using the pairwise comparison matrix, which compares the relative relevance of two criteria. As a result, the pairwise comparison matrix was created by taking into account the relative importance of each criterion and comparing them one-to-one using a pairwise scale [32,33]. The sub-criteria were weighted using Saaty's 1–9 scale [[19], [34]], with the greatest value (9) representing "extremely important" and the reciprocal (1/9) representing "extremely insignificant" (Table 4). The weights of each sub-criterion were determined using the logic outlined in Table 4. In this study, an 8x8-comparison matrix was performed by assigning a value from the range of 1–9 by comparing the parameter within the row to the parameter within the column (Table 6). For instance, from the matrix (Table 6), 9 was assigned for distance to water source compared to soil texture, soil depth, soil type, soil drainage, and LULC while 1/9 was assigned for soil texture, soil depth, soil type, soil drainage, and LULC compared to the distance to a water source.

**Table 4:** Fundamentals of Saaty scale (1980).

Scales	Preferential level	Explanation
1	Equally important	The parent decision element is influenced equally by two decision components.
3	Moderately more important	One decision factor has a moderately higher influence than the other does
5	Strongly important	One decision factor has a greater impact than the other does
7	Very strongly important	One decision factor has a lot greater influence than the other does
9	Extremely important	The contrast in influences between the two choice elements is enormous.
2, 4, 6, 8	Intermediate judgment values	Weights 1, 3, 5, 7, and 9 are used to illustrate compromises between preferences.

**Table 5:** Random index value By Saaty 1980.

n	1	2	3	4	5	6	7	8	9	10
RI	0.00	0.00	0.58	0.90	1.12	1.24	1.32	1.41	1.45	1.49

**Table 6:** Comparison matrix.

	Distance to Water source	Slope	Elevation	Soil texture	Soil drainage	Soil depth	LULC	Soil type
Distance to Water source	1	5	7	9	9	9	9	9
Slope	0.2	1	3	2	5	7	7	7
Elevation	0.14	0.33	1	2	7	5	5	5
Soil texture	0.11	0.5	0.5	1	5	7	7	7
Soil drainage	0.11	0.2	0.14	0.2	1	1	2	5
Soil depth	0.11	0.14	0.2	0.14	1	1	2	2
LULC	0.11	0.14	0.2	0.14	0.5	0.5	1	2
Soil type	0.11	0.14	0.2	0.14	0.2	0.5	0.5	1
Total	1.89	7.45	12.24	14.62	28.7	31	33.5	38
Number of comparisons = 28								
Consistency Ratio CR = 9.9%								
Principal Eigen value = 8.977								

#### 3.4.3 Evaluating consistency

Within the analytical hierarchy process, consistency of calculated weight for every parameter or criteria and sub criteria is one of the issues that require examination [31,32]. The consistency of the pairwise matrix is tested using a consistency ratio (CR) to check the degree of consistency or inconsistency once the judgments or weights of the criteria and sub criteria have been placed into the comparison matrix (Saaty1980, 1990; Garcí; a et al., 2014). The consistency index (CI) of the matrix is compared to the consistency of a random-like matrix using CR (RI). This will be determined using consistency ratio (CR) which may be estimated using equation (1).

(1) $$CR=\frac{CI}{RI}$$

(2) $$CI=\frac{\lambda_{\max}-n}{n-1}$$

CI is the consistency index (0.1396) which was calculated using equation (2) and RI is the random index value (Table 5). RI (1.41) is the average of the consistency index during which it depends on the matrix given by Ref. [19]; n may be a number of parameters (n = 8) and *λ*_max_ (8.977) is the principal Eigenvalue. CR is the consistency ratio, which refers to the degree of the consistency or inconsistency of the parameters or criteria [35]. When CR > 0.1, the pairwise comparison matrix is not correct and needs revision. Based on the result, the CR value for this study is < 0.1 which is CR = 0.098987, therefore the matrix is correct.

#### 3.4.4 Ranking and prioritizing the criteria

The values of each cell in the pairwise comparison matrix was divided by the total of its column. A "normalized matrix" is the output of this process [18]. The final priorities, denoted as "weights," were derived from this normalized matrix. As a result, the final AHP outputs are the relative priority of each criterion expressed in percentages, and (ii) a relative rank of each criterion.

### 3.5 Methodological summary

**Fig. 3:**
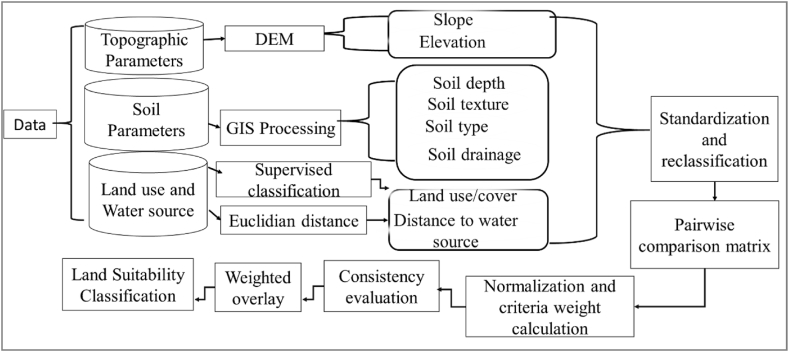
General methodology flow chart for this study.

Land suitability analysis is important to determine the foremost suitable areas for surface irrigation considering various factors. For this, the present study was conducted on two Districts/Woredas to determine the suitable farmlands for surface irrigation. In this study, an analytical hierarchy process method with GIS was used to evaluate the relationship between soil physical parameters, topography (slope and elevation), and land-use factors on the suitability of lands for surface irrigation. Data collection, satellite image analysis, land suitability factor evaluation, mapping, and GIS-based analytical hierarchy process land suitability modeling were applied. The input parameters were converted into Adindan UTM Zone 37 N projection system and the same pixel sizes (30 m × 30 m). Land suitability factors were rasterized, reclassified, and standardized based on the importance of every sub-criterion. After all, the suitability of the parameters was reclassified and rescaled using the weighted overlay method, which ranges from 1 to 9 [36]. The size of 1 refers to very low importance, but 9 is the higher importance. An evaluation of the relationship between land suitability and factors as well as the significance of every parameter and sub-criteria was performed using the principle of the analytical hierarchy process. Thus, the weight of every criterion was calculated employing a pairwise comparison matrix. The weighted parameters (criteria) were summed up using weighted overlay methods in ArcGIS to get a land suitability index as shown in Eq. (3), [37]. After all, the land suitability index map was classified into four suitability classes using the natural break method. These are highly suitable (S_1_), suitable (S_2_), marginally suitable (S_3_), and unsuitable class (S_4_). The general methodology flow chart for this study is summarized in Fig. 3.

(3) $$LSM=\sum_{i=1}^{n}W_{i}*X_{i}$$

Where LSM is the land suitability map, n is a number of criteria or parameters, Wi is criteria weight, and Xi is the criteria or parameter.

## 4 Results and discussion

### 4.1 Results

As shown in Table 6, the relationship between governing factors and land suitability for surface irrigation was determined using an 8 × 8 pairwise comparison matrix (Table 7). For this study, eight governing factors such as physical factors (soil texture, soil drainage, soil type, and soil depth), land use, topographic factors (slope and elevation), and distance to water source were considered. The weight of every factor was calculated from the normalized table by the average sum of the row of the matrix (Table 7). The relative significance of every factor is summarized in Table 7. Distance from a water source (44%), slope (18%), elevation (13%), soil texture (13%), and soil drainage (5%) have scored high weight criteria and they are the foremost important factors in determining suitable lands for surface irrigation (Table 7) followed by soil depth (4%), LULC (3%), and soil type (2%). In this investigation, the CR value was 0.1, indicating that there was no weight inconsistency. As shown in Table 7, the highest weight was given for distance from the water source, slope, elevation, soil texture, soil drainage, and soil depth due to the presence of low slope conditions, lands closer to rivers, good soil texture, and soil drainage.

**Table 7:** Normalized table.

	Distance to the water source	Slope	Elevation	texture	drainage	depth	LULC	type	Eigenvector of Weights	Weight %
Distance to a water source	0.53	0.67	0.57	0.62	0.31	0.29	0.27	0.24	0.44	44%
Slope	0.11	0.13	0.25	0.14	0.17	0.23	0.21	0.18	0.18	18%
Elevation	0.07	0.04	0.08	0.14	0.24	0.16	0.15	0.13	0.13	13%
texture	0.06	0.07	0.04	0.07	0.17	0.23	0.21	0.18	0.13	13%
drainage	0.06	0.03	0.01	0.01	0.03	0.03	0.06	0.13	0.05	5%
depth	0.06	0.02	0.02	0.01	0.03	0.03	0.06	0.05	0.04	4%
LULC	0.06	0.02	0.02	0.01	0.02	0.02	0.03	0.05	0.03	3%
Soil type	0.06	0.02	0.02	0.01	0.01	0.02	0.01	0.03	0.02	2%

### 4.2 2 Discussion

#### 4.2.1 Parameter suitability for surface irrigation

##### 4.2.1.1 Soil texture suitability

**Fig. 4:**
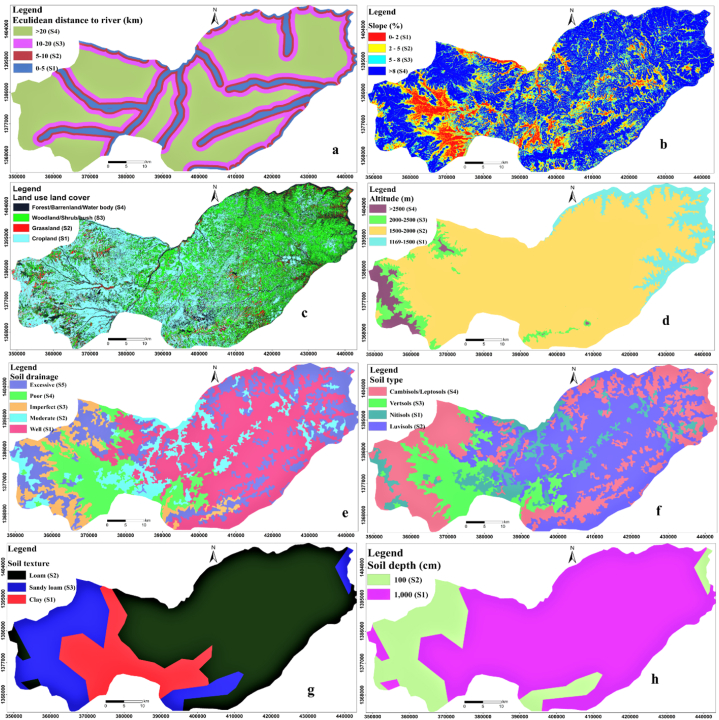
Land suitability factors/parameters a) distance to river b) slope c) LULC d) altitude e) soil drainage f) soil type g) soil texture and h) soil depth.

The study area is characterized by clay, loam, and sandy loam soil textures which range from highly suitable to a marginally suitable area for surface irrigation. Surface irrigation was deemed highly and moderately suitable for clay and loam textured soils, respectively [5]. Thus, clay texture soil was assigned a high relative influence value (Table 8). If the soil is characterized by a very coarse texture with very well sorted grain arrangements, the soil will lose the required water due to infiltration that might affect the moisture content that could be required for crop growth. However, if the soil is fine, it will keep moisture content in soil mass which could save time and water for irrigation. The result shows clay soil texture rated as highly suitable (S_1_-) and loam soil texture rated as moderately suitable (S_2_) which covered an area of 15.9%/433.0 km^2^ or 43296.5 ha and 62.5%/1697.6 km^2^ or 169763.6 ha (Table 8 and Fig. 4 g) of the study area, respectively. When compared to S_1_, the marginally suitable land class (S3- sandy loam) covers a moderately large area (approximately 21.6%/586.2 km^2^ or 58619.1 ha (Table 8) of the entire study area.

**Table 8:** Land suitability parameters class area summary.

Parameters	Parameter class	Land suitability	Pixel area (m^2^)	% Area	Area (km^2^)	Area (ha)
Soil drainage	Excessive	S5	758122	25.1	682.3	68231.0
	Poor	S4	393374	13.0	354.0	35403.7
	Imperfect	S3	231800	7.7	208.6	20862.0
	Moderate	S2	351087	11.6	316.0	31597.8
	Well	S1	1284480	42.5	1156.0	115603.2
Soil type	CM/LP	S4	989922	32.8	890.9	89093.0
	VR	S3	393374	13.0	354.0	35403.7
	NT	S1	351087	11.6	316.0	31597.8
	LV	S2	1284480	42.5	1156.0	115603.2
Elevation	>2500	S4	193544	6.4	174.2	17419.0
	2000–2500	S3	649723	21.5	584.8	58475.1
	1500–2000	S2	1464040	48.5	1317.6	131763.6
	1169–1500	S1	711360	23.6	640.2	64022.4
Distance to stream (km)	<5	S1	2002608	66.3	1802.3	180234.7
	5–10	S2	318065	10.5	286.3	28625.9
	10–20	S3	330580	11.0	297.5	29752.2
	>20	S4	367407	12.2	330.7	33066.6
Soil texture	Sandy loam	S3	651323	21.6	586.2	58619.1
	Loam	S2	1886262	62.5	1697.6	169763.6
	Clay	S1	481072	15.9	433.0	43296.5
Soil depth (cm)	10	S3	651323	21.6	586.2	58619.1
	100	S2	2367334	78.4	2130.6	213060.1
LULC	Forest/Barren land/Water body/Settlements	S4	748315	24.8	673.5	67348.4
	Woodland/Shrub/bush	S3	974974	32.3	877.5	87747.7
	Grassland	S2	125386	4.2	112.8	11284.7
	Cropland	S1	1170188	38.8	1053.2	105316.9
Slope (%)	<2	S1	96157	3.2	86.5	8654.1
	2–5	S2	382906	12.7	344.6	34461.5
	5–8	S3	331494	11.0	298.3	29834.5
	>8	S4	2208110	73.1	1987.3	198729.9

##### 4.2.1.2 Soil depth suitability

The Soil depth can influence agricultural production by determining the potential for rooting depth [5]. Surface irrigation become productive when the depth of soil in agricultural land is adequate. When the soil depth is shallow, crop root depth and soil water content are reduced, which can lead to crop failure in locations where frequent irrigation is not used. The soil depth of the study area ranges from moderately suitable to marginally suitable land classes (Table 8). The moderately deep soil profile has been rated as moderately suitable for surface irrigation, but a marginally suitable class for shallow soil profile (Table 8). The moderately suitable (S_2_) class is spatially distributed within the central part of the study area (Fig. 4h) and covered 78.4%/2130.6 km^2^ or 213060.1 ha (Table 8) of the study area. The marginally suitable land class (S_3_) is extremely shallow soil depth, which covered 21.6%/586.2 km^2^ or 213060.1 ha (Table 8) of the study area. It is spatially distributed within the south, the western, and eastern parts of the study area (Fig. 4 h).

##### 4.2.1.3 Soil drainage suitability

The soil characterized by well drainage is classified as a highly suitable land class (S_1_) because excess water standing on the land can affect the growing crops. S_1_ covered a relatively large portion of the study area. It covered 42.5%/1156 km^2^ or 115603.2 ha (Table 8) of the entire study area. Moderately suitable class (S_2_) is covered 11.6%/316 km^2^ or 31547.8 ha (Table 8 and Fig. 4 e) of the entire areas of the study. The marginally suitable land class (S_3_) is covered 7.7%/208.6 km^2^ or 20862 ha (Table 8) of the study area which covered relatively very small area compared to S1. Not suitable land class (S_4_) is covered 13%/354 km^2^ or 35403.7 ha (Table 8).

##### 4.2.1.4 Soil type suitability

Luvisols, Vertisols, Cambisols, Nitisols, and Leptosols are the principal soil categories found in the study area, as shown in Fig. 4f. Luvisols are fine to medium-grained clay-to-clay loam soils that are deep to extremely deep soils. Luvisols provide ideal conditions for surface irrigation systems in terms of all factors except that they are both limited by sandy loam (Girma and Tasisa 2020). In the study area, Luvisols (LV) covered a large portion of the study area (42.5%/1156.0 km^2^ or115603.2 ha) and it is classified as a moderately suitable (S_2_) class [11]; Girma and Tasisa 2020). Vertisols are soils with a high clay mineral content that shrink and swell as the water content changes. They expand when wet and shrink as they dry, causing deep fissures in horizons that extend at least 50 cm from the surface downward [11]; Girma and Tasisa 2020). They are deep to very deep soils that are poorly drained [11]; Girma and Tasisa, 2020). Vertisols are constrained by their poor drainage, but the other criteria are ideal for surface irrigation. As result, in this study, vertisols are classified as marginally suitable (S_3_) for surface irrigation that covers 13%/354 km^2^ or 35403.7 ha (Table 8) of the study area (Fig. 4 g). Cambisols are shallow to deep soils that are characterized by fairly, moderately well, and somewhat excessively drained. Cambisols in the lowlands are classed as Chromic, Calcaric, Leptic, and Vertic-calcic (OWWDSE 2010). However, in the study area, it is found on steep slopes and then classified as not suitable (S_4_) for surface irrigation due to it being found on steep slopes with rock fragments. The nitisols are found close to the river, which is classified as a suitable class (S_1_), and covered 11.6%/316.0 km^2^ or 31597.8 ha (Table 8) of the study area.

##### 4.2.1.5 Slope gradient suitability

In low slope-to-flat areas, surface irrigation was favored, while steep slopes were deemed inappropriate for surface irrigation [5]. The slope areas ranging from 0 to 2% were assigned high weights and classified as S_1_ class because the flat slope can allow water to effectively infiltrate into the soil to reach the crop root zone. But slope areas greater than 8% were considered unsuitable because the steep slope causes intensive runoff rather than reaching the crop root zone. According to the slope analysis, 26.9% of the flat lowland region is high to slightly appropriate for surface irrigation (Table 8). The slope of 73.1% of the study area is greater than 8%, making it marginally to permanently unsuitable for surface irrigation [[5], [11]]. A suitable range of slop classification for surface irrigation with miner modification to negotiate the natural slop was found in 26.9% of the study area [11]. The study region's slope suitability analysis found that 3.2% area, or 8654.1 ha, was in the extremely suitable (S_1_) range for surface irrigation. Similarly, the moderately suitable (S_2_) range for the surface irrigation system is found to be 12.7%/344.6 km^2^ or 34461.5 ha (Table 8 and Fig. 4 b) of the study area, while the remaining 11%/298.3 km^2^ or 29834.5 ha, and 73.1%/1987.3 km^2^ or 198729.9 ha of the total area of the Belessa were found to be marginally suitable (S_3_) and marginally not suitable (S_4_/N) with respect to the slope. As a result, 15% of the study area was deemed to be suitable for surface irrigation.

##### 4.2.1.6 Altitude/elevation suitability

The highly suitable land class of altitude/elevation covered 23.6%/640.2 km^2^ or 64022.4 ha (Table 8) of the entire study area, which is dominantly found within the north, and eastern parts of the study area (Fig. 3b). The moderate suitability class (S_2_) covered about 48.5%/1317.6 km^2^ or 131763.6 ha (Table 7), which is spatially distributed within the central part of the study area (Fig. 4 d). The marginally suitable land class (S_3_) is covered 21.5%/584.8 km^2^ or 58475.1 ha (Table 8), which is spatially distributed within the central, western, and southern parts of the study area (Fig. 3b). The 6.4%/174.2 km^2^ or 17419 ha (Table 8) is covered by unsuitable land class (S_4_), which is distributed within the western and south a part of the study area (Fig. 4 d).

##### 4.2.1.7 Land use/land cover suitability

Cultivated land was rated as very suitable (S_1_) for irrigation, based on the idea that these land cover types may be irrigated without restrictions (Girma and Tasisa 2020). The highly suitable land class (S_1_), covered 38.8%/1053.2 km^2^ or 105316.9 ha (Table 8) of the entire study area, but the second-class (S_2_) grassland covered 4.2%/1053.2 km^2^ or 105316.9 ha (Table 8) of the entire study area. The third land suitability class (S_3_) bush or shrub-covered 32.3%/877.5 km^2^ or 87747.7 ha (Table 8 and Fig. 4 c) of the entire study area. Other types of land, such as dense forestlands were considered unsuitable for irrigation. This is because the land used for these reasons cannot be put under cultivation, according to local culture (Girma and Tasisa 2020). Exposed rock surfaces with scattered shrubs or barren lands, water bodies, and settlement areas are not suitable to use for surface irrigation (S_4_). 24.8%/673.5 km^2^ or 67348.4 ha (Table 8) are covered by unsuitable land class (S_4_ or N_1_).

##### 4.2.1.8 Distance water source suitability

Distance from the water source is one of the most important parameters in land suitability analysis for surface irrigation which indicated the amount of land close to the water source (permanently flowing rivers). As the distance from the water source (permanently flowing rivers) increase, the accessibility for surface irrigation decrease, and the cost of operation will increase, therefore the close to a water source (permanently flowing rivers) is preferable for surface irrigation [2]. The distance between irrigable land and rivers in the study area ranges from 0 to 20 km. 68.2% (1802.3 km^2^ or 180234.7 ha) of the area is found as highly suitable lands (S_1_) for surface irrigation. 10.5% (286.3 km^2^ or 28625.9 ha), 11.0% (297.5 km^2^ or 29752.2 ha), and 12.2% (330.7 km^2^ or 33066.6 ha) of the areas are found as moderately suitable (S_2_), marginally suitable (S_3_) and not suitable (S_4_), respectively (Table 8 and Fig. 4 a).

#### 4.2.2 Land suitability map for surface irrigation

**Fig. 5:**
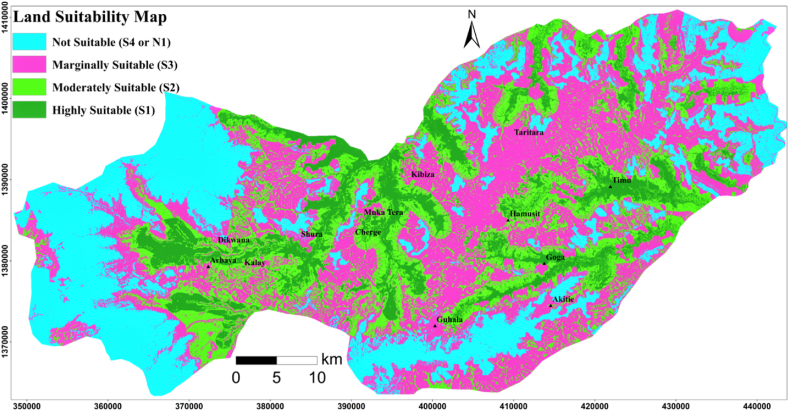
Land suitability map.

**Fig. 6:**
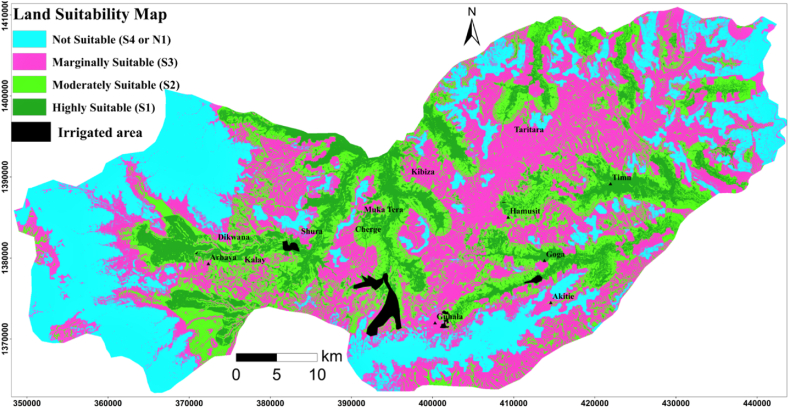
Land suitability map with irrigated land.

Land suitability analysis for surface irrigation required various input parameters including soils, LULC, distance from the water source (permanently flowing rivers), and topography. In this study, eight parameters were evaluated and weighted to determine the suitability of land for surface irrigation. Then, the weighted criteria maps were summed up together to quantify the land suitability map using the weighted sum method within the ArcGIS tool. The suitability map showed the spatial distribution of the suitable land classes for surface irrigation (Fig. 5). Out of the entire study area, 28.0% (761.6 km^2^ or 76164.7 ha) (Table 9) of the study area is covered by a not suitable area (S_4_) due to the far distance from the water source (permanently flowing rivers), steep slope, excessive drainage, shallow soil depth, high altitude, settlements, forest, waterbody, leptosols, and poor drainage [38]; Abraham et al., 2013; [3]. As indicated in Table 9, 37.6% (1020.3 km^2^ or 102033.3 ha) area was covered by a marginally suitable class (S3). Because, this region is characterized by imperfect soil drainage, moderately elevated area, marginally on the brink of a water body, sandy loam soil texture, very shallow soil depth, and bushland [38]; Abraham et al., 2013; [3]. 22.1%/598.9 km^2^ or 59890.7 ha (Table 9) area from the entire study area is covered by a moderately suitable class (S_2_). Due to the presence of moderately deep soil, grassland, moderately on the brink of a water source, nitisols, and moderately drained soil mass [38]; Abraham et al., 2013; [3]. The primary suitable class (S_1_) is covered 12.3%/334.7 km^2^ or/33466.7 ha (Table 9) of the entire study area due to the presence of well drainage soil, nitisols type, low elevated land, closest to water sources (permanently flowing rivers), clay soil texture, and currently cultivated lands. The central, northwestern, southwest, southeast, and northeastern parts of the study areas were found moderately to highly suitable lands for surface irrigation due to closer distance from the water source (permanently flowing river), flat slope, deep soil depth, well-drained soil, clay, and clay loam soil textures. Due to their steep slope (>8%), excessive soil drainage, shallow soil, long-distance, and sandy loam soil, the majority of unsuitable land is in the west, south, east, and some central areas of the study area, as shown in Fig. 5. Land suitability factors such as slope, soil depth, drainage, soil texture, soil type, and altitude were found to have a significant impact on the suitability of land for surface irrigation within the study area. As shown in Fig. 6, the accuracy of the land suitability map was evaluated by overlaying the existed irrigation which is fall under highly suitable and moderately suitable lands, which confirms that the present land suitability model is accurate.

**Table 9:** Land suitability class.

Land suitability class	Pixel Area (m^2^)	Area (%)	Area (km^2^)	Area (ha)
Not suitable (S_4_)	846274	28.0	761.6	76164.7
Marginally suitable (S_3_)	1133703	37.6	1020.3	102033.3
Moderately suitable (S_2_)	665452	22.1	598.9	59890.7
Highly suitable (S_1_)	371852	12.3	334.7	33466.7

## 5 Conclusion

In the present study, eight parameters were evaluated using the rule of the analytical hierarchy process method integrated with ArcGIS to produce a land suitability map for surface irrigation. The results of the pairwise comparison matrix from the normalized table showed that distance from a water source (44%), slope (18%), elevation (13%), soil texture (13%), and soil drainage (5%) have scored high weight criteria. Moreover, they are the foremost important factors in determining suitable lands for surface irrigation (Table 6) followed by soil depth (4%), LULC (3%), and soil type (2%). After the weight of every parameter was calculated, the final suitable map was generated with four classes of highly suitable (S_1_), moderately suitable (S_2_), marginally suitable (S_3_), and not suitable (S_4_). The result showed that 12.3%, 22.1%, 37.6%, and 28.0% of the study area is covered by highly suitable (S_1_), moderately suitable (S_2_), marginally suitable (S_3_), and not suitable (S_4_), respectively. Hence, this work showed the spatial distribution of suitable and not suitable lands for surface irrigation, the concerned body could also be at the Zonal/Woreda level should take a measurement to implement surface irrigation in highly suitable regions (S_1_) and moderately suitable (S_2_). Hence, this work is only focused on the assessments of the suitability of the land for surface irrigation; it can be recommended that evaluate the availability of water, suitability of chemical properties of soil, and social and economic parameters in a region for surface irrigation.

## Author contribution statement

Azemeraw Wubalem: Conceived and designed the experiments; Performed the experiments; Analyzed and interpreted the data; Contributed reagents, materials, analysis tools or data; Wrote the paper.

## Funding statement

This research did not receive any specific grant from funding agencies in the public, commercial, or not-for-profit sectors.

## Data availability statement

Data will be made available on request.

## Declaration of interest’s statement

The authors declare no conflict of interest.
